# Building a diverse and inclusive plant science community

**DOI:** 10.1093/jxb/erag060

**Published:** 2026-02-07

**Authors:** Andrea Paterlini, Agnes Uhereczky, Yoselin Benitez-Alfonso, Sofie Goormachtig, Devang Mehta, Mary E Williams

**Affiliations:** Institute of Molecular Plant Sciences, School of Biological Sciences, University of Edinburgh, Edinburgh EH3 9LL, UK; Vlaams Instituut Voor Biotechnologie (VIB), Ghent, Belgium; Centre for Plant Sciences, School of Biology, Faculty of Biological Sciences, University of Leeds, Leeds, UK; Department of Plant Biotechnology and Bioinformatics, Ghent University, Ghent, Belgium; Center for Plant Systems Biology, VIB, Ghent, Belgium; Department of Biosystems, KU Leuven, Leuven, Belgium; Leuven Institute for Single Cell Omics, KU Leuven, Leuven, Belgium; American Society of Plant Biologists, Rockville, MD, USA; Max Planck Institute for Molecular Plant Physiology, Germany

**Keywords:** Diversity, equity, inclusion, mentoring, plant science, research culture, teaching

## Abstract

For many people, the culture of plant science, and science more broadly, can feel alienating and intimidating, which often leads to them leaving the discipline for other opportunities. However, studies show that there is a correlation between creative problem solving and increased diversity, and plant science cannot afford to lose talented individuals. Here we report on strategies to promote diversity, inclusion, and a sense of belonging for all within plant science. We address ways that institutions, organizations, communities, educators, and individuals can contribute to this needed cultural change. We urge all plant scientists to participate in these efforts; for each other, for the discipline, and for the future.

## Teaser

This article highlights strategies being used by various stakeholders (institutions, professional societies, community groups, educators, and individuals) to promote a plant science culture that is welcoming to all.

## Introduction

Today’s culture of science is built upon structures that were formulated hundreds of years ago, mainly in Europe and more recently also in the USA, by a relatively homogenous group of power holders, namely white men. Scholars have gone as far as to coin the expression ‘Science as White property’ (Mensah and Jackson, 2018). Although the practitioners of science have diversified, representation still lags behind current demographics in these same regions (Bhalla *et al*., 2025). Although many factors contribute to this skewed representation, the culture of science—including its persistent biases against women, ethnic minorities, people with disabilities, and members of the LBGTQ+community—is an important contributor (Asai, 2020; Eaton *et al*., 2020; McGee, 2020; Cech, 2022; National Academies of Sciences, Engineering and Medicine, 2023). Furthermore, geographical and economic biases provide additional layers of exclusion (Khelifa and Mahdjoub, 2022). Berhe *et al*. (2022) eloquently describe the path of scientists from historically excluded groups as a ‘hostile obstacle course’ In recent years, many studies have been published advocating for specific changes to support all members of the scientific community and create a culture in which all can feel that they belong (e.g. Willis *et al*., 2020; Arif *et al*., 2021; Field and Rajewski, 2021; Henkhaus *et al*., 2022; Peña *et al*., 2022; Ruedas-Gracia *et al*., 2022; Puig-Lluch *et al*., 2025).

The authors of this article led a workshop on the topic of ‘Building a Diverse and Inclusive Community through Effective Teaching and Mentoring’ at the International Conference on Arabidopsis Research (ICAR) in June 2025 in Ghent, Belgium. The goal of this workshop was to raise awareness of the persistent and systemic biases within science that disproportionally affect people historically excluded by race, gender, disability, and other marginalized identities, and to highlight ways in which these biases can be mitigated through institutional, organizational, community, and individual actions, as well as actions that can be employed specifically in the teaching and learning environment. These discussions are timely, given explicit and implicit attacks on diversity, equity, and inclusion (DEI) efforts in the USA and other countries (Bhalla *et al*, 2025; Lakhani, 2025).

Here we present a summary of the workshop and highlight concrete steps that can be implemented at various organizational levels to promote inclusion in plant science. We acknowledge our specific positionality: we are professionals based in the UK/Europe with some experience of other research environments. We hope that the issues identified and recommendations provided can still be of broad relevance. The range of topics discussed was also constrained by the duration of the workshop itself.

## Understanding and dismantling barriers to inclusion

**Fig. 1.**
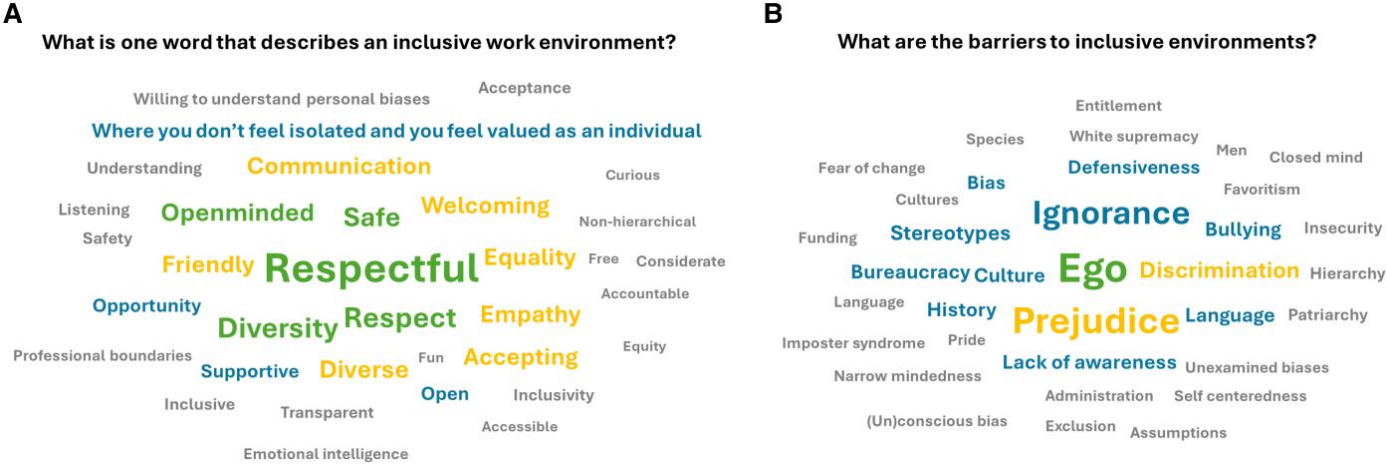
Audience input on the topic of inclusive working environments. (A and B) Wordclouds were generated from submissions made by workshop attendees (via Slido live polling). The size of each word is proportional to the number of entries received listing that word. The first word cloud (A) displays terms attendees associated with inclusive working environments, while the second (B) captures perceived barriers to achieve such spaces.

Conversations about bias and discrimination can be challenging and uncomfortable, so we started the workshop by prompting attendees to reflect on what factors describe an inclusive environment (Fig. 1A) and what are the major barriers to achieving such an environment (Fig. 1B). We used an anonymous online tool to collect inputs from our audience, ∼50 conference delegates, which spanned different genders, career stages, geographical locations, and backgrounds. Our audience was a subset of the 600 in-person attendees present at the conference, 40% of which self-identified as early career researchers. Nearly 50 countries were represented at ICAR 2025.

The responses to the prompts indicate that respect and safety were prominently associated with inclusive environments, as has been well documented (Royal Society of Chemistry, 2021; Ruedas-Gracia *et al*., 2022; National Academies of Science, Engineering and Medicine, 2023). Building on that, a generally welcoming environment was sought. Themes probably relating to personal identity also featured, accompanied by core concepts of diversity and equity. Inter-personal communication and understanding were also valued. Lastly, accountability, transparency, and power dynamics were also highlighted.

The ego of individuals within the community was perceived as a key barrier hindering inclusive environments (based on responses of participants). Entitlement and pride were also noted, alongside historical, cultural, and colonial legacies (with lingering ethnic- and gender-based hierarchical structures). Misplaced perceptions that equate brilliance and bravado, the latter degenerating into masculinity contests, probably reinforce and perpetuate these attitudes in Science (Berdahl *et al*., 2018; Glick *et al*., 2018; Vial *et al*., 2022). A number of terms also pointed to a widespread lack of awareness around diversity. Inappropriate, exclusionary, or discriminatory behaviours were also listed. Lastly, existing funding and administrative frameworks were also deemed problematic.

These goals and limitations are clearly not exhaustive but highlight both a desire for change and a need to address multiple existing barriers. It is perhaps not surprising that, overall, there was broad alignment between audience responses and topics discussed in the literature (Emery *et al*., 2021; Henkhaus *et al*., 2022).

We followed this initial activity by watching a short video in which professional actors portrayed an incident of harassment in a research workplace (VIB Code of Conduct eLearning internal video). The video opened with a casual conversation between two research group members (a research technician and a postdoctoral researcher) in a shared office space. This interaction was abruptly interrupted by the research group leader storming into the room and aggressively accusing the technician of not having performed an experiment according to the requested specifics. The group leader went on to call into question the skills of the individual and concluded that they were going to take care of the experiment themselves, as they had no trust in the technician. A key feature of the video was that the other group member present (the postdoctoral researcher) remained quiet throughout the exchange.

**Fig. 2.**
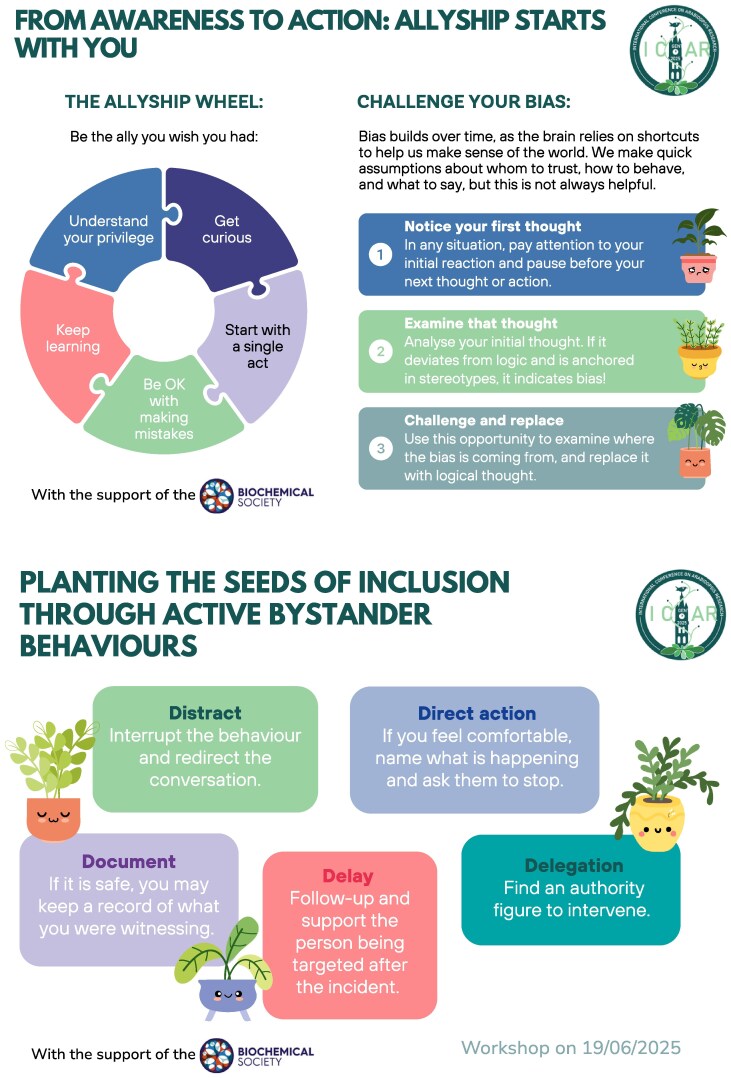
Postcard handed out to support reflection and audience engagement. Individual workshop attendees were provided with a copy of this resource. One side of the postcard explores how to be an effective ally and challenge our biases. A video portraying a (rehearsed) instance of harassment in a research setting was displayed in the workshop. The other side of the postcard lists effective interventions (Distract, Direct Action, Document, Delay, and Delegation) that can be made by active bystanders to address such a situation and support the person being harassed. The postcard was designed by Agnes Uhereczky.

We provided workshop participants with postcards outlining possible actions that could have instead been taken to address the inappropriate behaviour and support the person being harmed (Fig. 2). These behaviours map onto the concept of ‘active bystander’ (Right To Be, 2024): someone who witnesses a potentially harmful situation and chooses to intervene, rather than passively observing. We asked attendees to imagine themselves in the situation portrayed and identify an effective action among the possibilities listed (Fig. 2). The purpose of this exercise was to highlight that everyone has an opportunity to contribute to a more inclusive environment. The other side of the postcard highlights additional steps individuals can take to reflect on their own biases and act as an ally to others (Fig. 2).

**Fig. 3.**
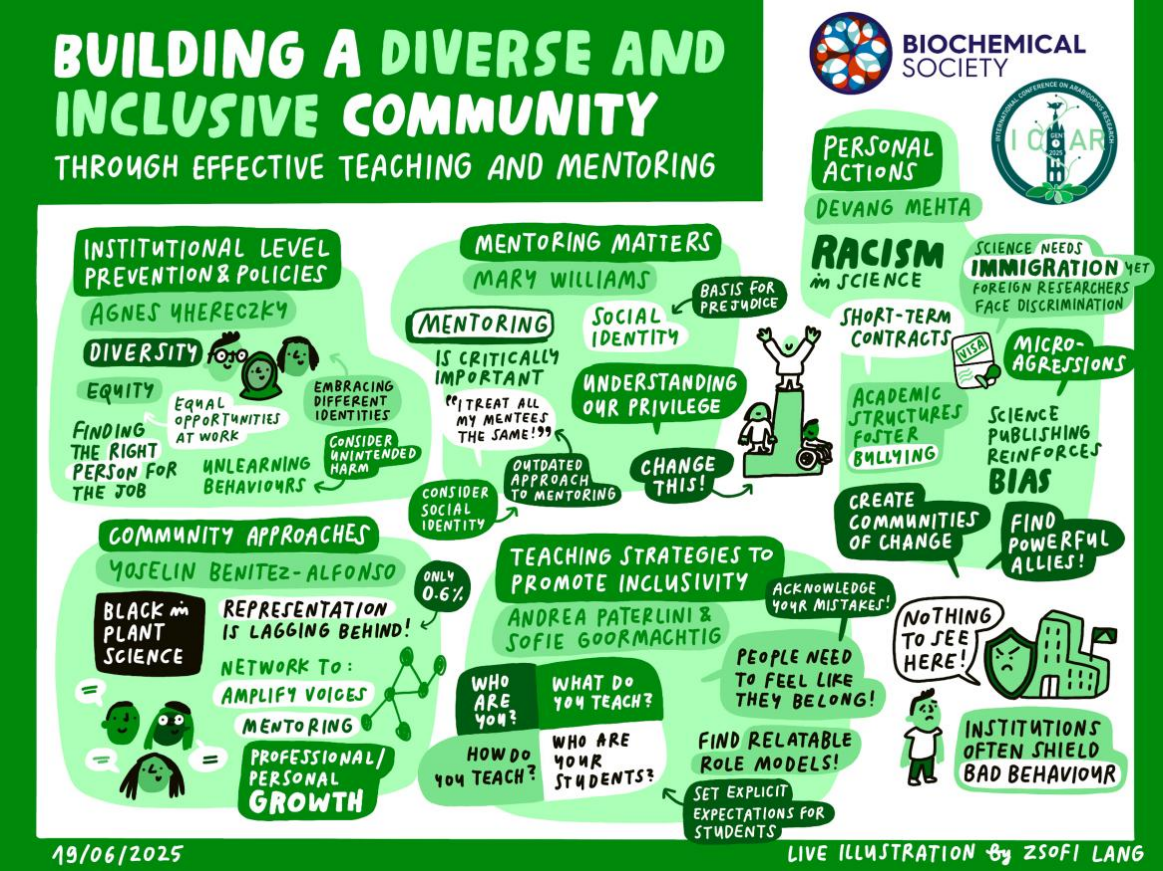
Graphic recording of the workshop session. Key words, messages, and topics covered are grouped by speaker(s). This visual summary is intended to capture the essence of the session, it obviously cannot resolve or explore in detail all the complex issues listed. The illustration was commissioned from the illustrator Zsofi Lang.

The remainder of the workshop featured five short presentations from the authors of this article, summarized below (and graphically in Fig. 3), with the goal of disseminating awareness of positive actions to the broader community.

## Bridging policy and impact: a framework for institutional diversity, equity, and inclusion

DEI have become strategic imperatives for institutions seeking to build resilient, innovative, and socially responsible cultures. Yet despite increased attention and effort, systemic barriers continue to limit the full participation and recognition of under-represented groups. To drive meaningful progress, DEI must evolve beyond performative statements into integrated, data-informed, and behaviourally anchored strategies, such as those laid out in the National Academies report on advancing anti-racism in Science, Technology, Engineering, and Mathematics—STEM (National Academies of Science, Engineering and Medicine, 2023).

At the heart of this transformation lies a clear understanding of the DEI continuum. Diversity ensures representation across dimensions such as race, gender, age, neurodiversity, and cultural background. Equity goes a step further—ensuring that opportunities, resources, and support structures are accessible to all, tailored to individual needs. Inclusion builds the cultural foundation for engagement, while belonging reflects the psychological safety people experience when they can be their authentic selves in the workplace (Zheng, 2022).

Institutional change requires simultaneous action across several pillars (Griffin, 2020). Policies and initiatives—such as anti-bias training, employee networks, allyship programmes, and confidential reporting mechanisms—are crucial. However, these must be coupled with active process reviews in recruitment, onboarding, performance evaluation, and leadership development to dismantle structural inequities and to truly embed inclusion and equal opportunities deep into the inner workings of the institutions. As examples, numerous programmes and outcomes stemming from the DEI efforts at the VIB (Vlaams Instituut voor Biotechnologie) can be found on their website (https://vib.be/en/careers/diversity-equity-inclusion-vib#/).

One of the most compelling arguments for this approach comes from a growing body of research linking diversity to innovation. Hofstra and colleagues identified a phenomenon known as the ‘diversity–innovation paradox’, wherein under-represented researchers in science consistently produce more novel contributions but receive less recognition and fewer rewards than their majority-group peers. This paradox highlights a persistent gap between talent and institutional support—diversity alone is not sufficient if systems fail to value and reward difference equitably (Garcia Martinez *et al*., 2017; Hofstra *et al*., 2020).

While diversity fuels recombination and holds great potential for innovation, it can simultaneously create challenges related to coordination, communication, and cohesion (Garcia Martinez *et al*., 2017; Smith-Doerr *et al*, 2017). As diversity can also lead to more conflict, research institutions need to foster an intellectually and socially safe environment for conflict to always remain unbiased and constructive, to allow tapping into the ‘collective brain’ (Schimmelpfennig *et al*., 2021).

This reinforces the need for behavioural and cultural change within institutions. Inclusive leadership, courageous conversations, and active bystander programmes help build the cultural scaffolding that allows diverse perspectives to be heard and valued (Willis *et al*., 2020). Institutions must foster an environment where disagreement is safe, feedback is welcomed, and divergent ideas are encouraged (National Academies of Science, Engineering and Medicine, 2020).

Data collection is also critical. Just as the study by Hofstra *et al*. (2020) used large-scale data to reveal hidden disparities, organizations must collect both quantitative and qualitative insights to monitor equity gaps, guide interventions, and measure progress. Transparency and regular reporting build accountability and trust among employees.

A useful way to measure the progress of individual institutions is to chart the evolution of the work on a DEI maturity model—from compliance-based approaches, to managing and supporting diversity, and ultimately to leveraging it as a strategic asset (Washington, 2022). At higher levels of maturity, institutions actively remove barriers to participation, empower under-represented voices, and integrate inclusion into their organizational DNA.

In sum, advancing DEI requires sustained effort, honest reflection, and a willingness to shift power. Institutions must align their policies, practices, and people strategies to foster a culture where diverse talent is not only present but also supported, celebrated, and elevated (Martinez-Acosta and Favero, 2018; Griffin, 2020). The rewards are profound: a workplace where all individuals belong, and where the collective power of difference drives research and scientific excellence and innovation (Wong *et al*., 2022; Asai, 2025).

Specific actions and efforts should focus on the following.

- Endorsement and sponsorship is needed from senior leadership within academic and scientific institutions, who understand not only the opportunities, but also the challenges associated with investing in DEI work.
- Institutions need to dedicate resources to managing DEI and not only focusing on the individuals, but on institutional processes, practices, and culture to foster inclusion and belonging. Resources may include a dedicated DEI manager, or a committee responsible for inclusion (with a clear mandate and authority).
- Support is needed for the creation of Employee Resource Groups to foster belonging, peer support, and advocacy for under-represented groups and allow them to create a safe space where they can be with peers sharing the same lived experience.
- Speaking up should be encouraged by providing safe reporting mechanisms that protect confidentiality and addressing issues of bias, harassment, or discrimination promptly and transparently, protecting against retaliation.

## Harnessing the power of professional societies to develop a cross-organizational mentor training programme

As voluntary communities of shared interest that cross institutional and career stage boundaries, professional societies have a special opportunity to promote diversity and inclusion within a discipline (Madzima and MacIntosh, 2021; Meyer-Gutbrod *et al*., 2023; Segarra and Etson, 2023; Ryan *et al*., 2025). In 2021, a group of several plant science professional societies was awarded a Research Coordination Network grant from the National Science Foundation to lead cultural change in the plant sciences, ROOT & SHOOT (Rooting Out Oppression Together and SHaring Our Outcomes Transparently; DBI-2134321). One of its goals is to develop an equity-based mentor training programme leading to cultural change that supports the advancement of individuals from all social identities. In the ICAR 2025 workshop, we reported on the goals and development of the Social Identity Matters In (SI-MI ‘see me’) Mentoring initiative.

The academic research environment relies on one-on-one mentoring relationships, through which early-career scientists refine skills and develop their identity as a professional (Gaut, 2025). Good mentoring interactions lead to measurably better outcomes, whereas bad ones contribute to attrition from the pipeline (National Academies of Science, Engineering and Medicine, 2019; Malone and Harper, 2022). Many mentor training programmes overlook the importance of cultural awareness and consideration of social identities. The Center for Improvement of Mentored Experiences in Research (CIMER) has developed programmes and resources to address this deficiency (Byars-Winston *et al*, 2018; Womack *et al*., 2020), and ROOT & SHOOT has partnered with CIMER to create a free training for plant scientists.

Social identities are categories such as race, ethnicity, disability status, or gender, based on shared social constructs, and are often at the root of unequal power and privilege. For example, within an academic context, students whose parents completed college have more access to information and norms that are often hidden from first-generation college students, contributing to improved outcomes (Hopkins *et al*., 2024). People with social identities that align with the mental image that many people have when they imagine a scientist (white, male) face less bias and discrimination (Berhe *et al*., 2022). The SI-MI Mentoring programme helps mentors to recognize that social identities have real impacts, and to reflect on how their journey and those of their mentees has been impacted by their social identities.

When advising students from sociodemographic groups that have been historically excluded from science, some mentors fall back on a deficit model of thinking; they assume that these students are less likely to succeed, which contributes to the mentees’ perception that they do not belong (Yosso, 2005; Valencia, 2010; Estrada *et al*., 2016; Reyes and Duran, 2021). SI-MI Mentoring advocates that mentors adopt a growth mindset; just as we seek to identify which aspects of the environment are hindering growth of our plant, mentors should explore and address the systemic obstacles that hinder progress of their mentees (Montgomery, 2018).

Through their actions and leadership, professional societies can reach out to departments and programmes that have direct leverage over mentors. We encourage all institutions to provide support, incentives, and accountability to promote effective mentorship (Montgomery *et al*., 2022). Even without such incentives, motivated mentors can challenge themselves to develop mentoring skills, for example through the resources at CIMER, online guides such as provided in White *et al*. (2018), and the SI-MI Mentoring programme. The next generation of plant scientists is counting on us.

Specific actions and efforts should focus on the following.

- Professional societies and organizations can promote and encourage their members to participate in professional mentorship training.
- Professional societies and organizations can influence the culture of their discipline through advocating for better practices within academia and industry.
- Organizations should commit to diversity and inclusion in all their activities, and hold themselves accountable by reporting on their progress.

## Community initiatives to empower under-represented backgrounds: examples from building ‘Black in Plant Science’

Researchers from Black and other under-represented backgrounds often report feelings of isolation, experience both micro- and macroaggressions, have their lived experiences denied, and regularly contend with assumptions of positive discrimination (Garrett, 2024). Even when their work is recognized, Black leaders frequently face increased (and often unpaid) workloads due to participation in multiple committees and panels, as well as the expectation to represent their communities. These challenges hinder their potential for further success and advancement.

Despite recent UK efforts to improve diversity in STEM, only 0.6% of full UK science professors identify as Black (Gibney, 2022). In contrast, 11 UK universities report >20% of their student population as being of Black heritage. Hubbard *et al*. (2024) reveal a troubling trend for UK plant science: hardly any home undergraduate students pursuing plant science degrees identify as Black. Students of Asian and Mixed heritage backgrounds are also under-represented in UK plant sciences, albeit to a lesser extent. These disparities inspired a team of two Professors, one Associate Professor, one industrial researcher, two postdoctoral researchers, and a PhD student to establish the Black in Plant Science (BiPS) Network in 2023. This UK-based community aims to connect, celebrate, and support Black plant scientists. Its mission is to amplify voices, validate the work of Black researchers, foster a sense of belonging, and build self-confidence. Similar organizations might already exist in other countries. BiPS is used here as a positive example to encourage the development of similar networks elsewhere.

BIPS provides sponsorship opportunities through awards, research studentships, and conference travel grants. To financially support and promote these activities, BiPS collaborates with diverse organizations: the North American Arabidopsis Steering Committee (NAASC), the Society of Experimental Biology, the *Plant Journal*, the New Phytologist Foundation, the University of Leeds, Bioimaging UK, the Grow More Foundation, and many others. As of the beginning of 2026, four Research Excellence Awards, six undergraduate summer studentships, and >20 travel awards for national and international events have been awarded to UK-based students/early career researchers of Black heritage.

In 2024, grant funding from the Gatsby Foundation supported a community network coordinator, significantly expanding the capacity of BiPS. The network has been able to launch a website (https://blackinplantscience.org/) featuring member profiles, DEI-related literature, and resources to increase visibility and representation. BiPS can now also promote activities and celebrate community achievements through its social media channels (LinkedIn, Bluesky, and Instagram). It also collects feedback and new ideas using community surveys or direct messages.

BiPS has also hosted online and in-person community events. The Black in Plant Science yearly conference, on the other hand, aims to spotlight diversity in plant science and bring together speakers from around the world. All BiPS events are designed to be inclusive and accessible, promoting the exchange of ideas/experiences between academics from different disciplines, funders, and researchers in industry. They also create opportunities for celebration and networking. More details on the 2025 conference are available online. The events are being attended by an increasing number of participants (>80 for the 2025 conference) and ∼25% of these attendees are not Black, indicating important awareness and allyship within the broader plant science community.

The impact of BiPS activities has been significant: students reported an improved sense of belonging and connection after meeting at conference events, while awardees experienced career progression and new opportunities thanks to enhanced visibility.

Lastly, one of the key challenges faced in research is mentoring across cultures. Inclusive leadership requires an understanding of cultural dynamics, which can influence project success and foster environments where everyone—regardless of ethnicity—feels empowered to contribute (Cox *et al*., 2021). Members of the BiPS committee received UK Research and Innovation funding to organize a culturally sensitive mentoring workshop. The workshop brought together mentors and mentees from diverse backgrounds and resulted in significant improvements in recognizing unconscious bias and developing culturally competent mentorship practice.s. It also highlighted institutional and cultural barriers, the importance of demographic considerations in mentor–mentee matching, and the elements necessary for building trust in mentoring relationships.

These initiatives are just some examples of the many ways in which BiPS is working to change the status quo by creating resources and opportunities to recruit, support, and retain Black plant scientists in the UK.

Specific actions and efforts should focus on the following.

- Raising awareness of the historical and systemic barriers limiting the success and recruitment of plant scientists from minoritized backgrounds.
- Iimplementing supportive systems for early-career researchers, including effective mentorship strategies.
- Building on the experience and integrate efforts across community networks such as BiPS and Women in Crop Sciences.
- Recognizing and investing in community networks, encouraging participation and support from funders. These networks benefit not only minoritized plant scientists but the broader scientific community, as diverse teams are proven to drive higher innovation and impact.

## Striving for inclusive education in our classrooms

Inclusive education broadly refers to efforts and approaches that consider the varying needs of students and that support all learners to participate and achieve the best possible outcomes (Hubbard and Gawthorpe, 2024). Recruitment and retention within STEM can benefit from inclusive education: a more diverse and creative pool of students might ultimately reach professional careers. Given the representation disparities in the sector (Marks *et al*., 2023; Hubbard *et al*., 2024), there is a broad need to reconsider plant science education as a whole (Alvey *et al*., 2025).

Concrete actions for inclusive education should respond to specific historical, institutional, disciplinary, and societal contexts. A reflective framework can help practitioners identify local challenges and opportunities. Marchesani and Adams (1992), for example, encourage educators to reflect on their lived experiences; on those of their students; on their subject matter; and on the pedagogical approach they use. As an example, authors recently reflected on the use of gender-connotated terms to talk about plant reproduction and offered more inclusive alternatives (Subramaniam and Bartlett, 2023). This personal reflection is the first step in a model that should also foster belonging in classrooms; set explicit student expectations; recognize diversity and address barriers to inclusion; and strive to design for accessibility (Appert *et al*., 2018; Dewsbury and Brame, 2019; Hales, 2020; Hubbard and Gawthorpe, 2024).

Inclusive education is to be viewed as an ongoing process, rather than an off-the-shelf didactic approach. It responds to societal changes and is driven by iterative reflection and adjustment, robustly supported by the evolving pedagogical literature. The ‘Black Lives Matter’ movement (and the tragic injustice episodes it stemmed from), for example, motivated many university instructors to explicitly incorporate race, racism, and racial equity content in their curricula (Scheuermann, *et al*., 2024). Inclusive education is therefore a journey of self-development for educators as much as it is for the students (Dewsbury and Brame, 2019). Practitioners must be open to this, also acknowledging and learning from unintended missteps.

Interventions aimed at diversifying the curriculum (by including a wider range of perspectives and figures) may appear superficially simple. Gladstone and Cimpian (2021) identify that the portrayal of science role models as meaningfully close to the students is instead critical for their effectiveness. Similarities should span both demographic and psychological factors. The careers displayed should also feel attainable: analogous paths to personal and professional fulfilment must be envisageable by the students. Highlighting diverse routes to make positive impacts on societies is also important to dispel skewed portrayals of success (Davies *et al*., 2021). Failing to consider these aspects might result in students feeling demotivated, rather than inspired.

As educators, we must also openly address historical biases in science (Joshi *et al*., 2024). The process of decolonization focuses on legacies of colonialism and marginalization in our science. Confronting these topics can be challenging, but is fundamental to ensure our students are informed agents of modern societies (Mansfield *et al*., 2024). This is particularly critical for botany (and, by extension, molecular plant science) as the collection and characterization of plant species has extensively relied on indigenous knowledge, largely without due credit (Mabry *et al*., 2024). We should introduce students to initiatives such as the Nagoya Protocol (Marden *et al*, 2021) and to debates around successes/failures in applying this benefit-sharing agreement for plant materials (Bennett *et al*., 2024; Kloppenburg *et al*., 2024). It is also important to stress that colonialism is not a feature of the past: modern economic and political practices continue to perpetuate similar dynamics (Andrews, 2021).

In addition to a reflection on lingering or new disparities, at the heart of inclusive education also lies a profound acknowledgement of the individuality of learners (and educators alike) (Felder and Brent, 2013). Socioeconomic backgrounds (e.g. the need to work while studying) and caring responsibilities (with different cultural expectations in that respect) are two examples of factors that can profoundly modulate educational experiences (Crockford *et al*., 2015; Spacey *et al*., 2024). Assessment, engagement, and support should be designed with equity, rather than equality, in mind (Elgin *et al*., 2021, Preprint; Handelsman *et al*, 2022). The situation of a person might also change with time, highlighting a need for dynamic considerations. At times, these goals may clash with logistical challenges experienced by educators. Structural solutions to dismantle barriers may require a profound rethinking of higher education. Nonetheless, we can begin by charting helpful paths across the complex landscape navigated by our diverse students. The value of being our authentic selves (Brookfield, 2015) and demonstrating kindness and understanding should not be underestimated (Arif *et al*., 2021).

Specific actions and efforts should focus on the following.

- Educators should critically examine their own context in relation to diversity and inclusion, considering how their personal beliefs and potential biases influence their teaching practices.
- Educators should regularly reflect on the challenges and opportunities experienced by students in their courses, considering if the same can be adjusted to equitably improve outcomes.
- Efforts to diversify and decolonize the curriculum need to be carefully considered if they are to improve the sense of belonging and empowerment of all students.

## Personal actions to drive change and inspire others

In addition to institutional and societal efforts to promote diversity and inclusion, the power of individual actions to catalyse change and inspire others should not be understated. A particularly potent such action is the sharing of personal experiences by scientists from marginalized and minoritized groups through editorials, in conference talks, and at other venues (Mehta, 2018; Desai *et al*., 2021). In an environment where the voices of marginalized scientists are not often heard, this can stimulate both other members of the marginalized groups and also the wider scientific community, which is often unaware of the challenges faced by the former. A powerful example of such communication is the book ‘*The autobiography of a transgender scientist*’ by the late neuroscientist Professor Ben Barres, which testified to the prevailing transphobia and sexism endemic in wider society and mirrored in the scientific community (Barres, 2018). Although academia today offers some channels for individuals to publicly speak up and share their experiences, such contributions are often discounted in professional evaluations of academic work (highlighting the need for merit review criteria to be re-evaluated). It is critical that when minority scientists take on the risk of sharing their personal stories, exposing themselves to personal attack, these efforts are supported and encouraged by the wider community and by existing organizations (as an example, see Nature Communications, 2022).

While highly effective, individual actions to promote diversity and inclusion are also very time-consuming and emotionally taxing. Hence, minoritized scientists must be allowed to focus their efforts on issues close to their hearts and avoid being tasked with excessive service work by their organizations, as is often the case (Järvinen and Mik-Meyer, 2024). Furthermore, individual action should be leveraged to create communities of change which can work to create institutional reform. During the workshop, the example of the Early Career Advisory Group (ECAG) at the journal *eLife* was discussed. The ECAG is a gender-diverse group of early career scientists from different geographical backgrounds that together tackle issues such as publication bias through various initiatives at the journal and the broader community (Kent *et al*., 2022). Thus far, the ECAG has united scientists from both marginalized and majority groups in science to support a variety of positive changes (Mehta *et al*., 2020), including the rapid diversification of the Editorial Board at the journal through targeted ‘Open Calls’ for new editors, a first at any major journal (see eLife, 2023). Following this example, many academic journals have instituted similar advisory boards to help drive change, offering successive generations of minoritized scientists an avenue to improve the status quo.

For individuals from minoritized groups, it remains true that efforts to enact institutional change can seem extremely slow and sometimes dispiriting. However, in our experience, working within our scientific institutions is a surer path to success than the oft-tempting route of castigating the system from outside. This can take the form of participating in various committees in our home institutions, or in our scientific societies or journals. For example, in many institutions, tenure and promotion committees can often have enormous impact on the diversity of senior professors, and membership in these by scientists from marginalized groups can be an effective channel to drive lasting change (Eaton *et al*., 2020; Masters-Waage *et al*., 2024).

Finally, speaking up and taking individual action is not without risk of both personal and professional attacks, from social media to anonymous reviewer comments in grant proposals (personal experiences of the authors). It is hence important that highly reputed scientists in the field speak up and demonstrate allyship with scientists from marginalized communities and thus establish norms and standards that welcome minoritized voices.

Specific actions and efforts should focus on the following.

- Minority and marginalized scientists should share personal experiences and challenges faced in order to spotlight areas of improvement and create deeper awareness of the toll caused by bias and discrimination.
- Scientists taking individual actions should also come together to create communities of change that can drive institutional reform.
- Professional societies, host institutions, and reputed leaders in the scientific community should step up and demonstrate allyship and create a platform for marginalized scientists taking individual action to promote diversity and inclusion.

## Conclusion

A diverse and inclusive plant science community can and will be achieved. To sustain and grow the discipline, we must manifest and actively strive for this goal. The path needed for this substantial change, however, remains long and will require sustained efforts. Resistance to change and slow movement are characteristic of the complex institutions we inhabit—be they higher education providers or research institutes (Rosenberg, 2023). We must not be discouraged by this.

The agency and capacity of individuals in our communities to act, for example, should not be underestimated. We can all engage in concrete actions. By analogy with an emerging research topic in plant biology, each attendee at our workshop (or reader of this article) is now a nucleating agent. They carry the potential—and perhaps the responsibility—to aggregate within their own institutions and communities other individuals who are open to exploring these important concepts. By priming formal and informal conversations, we can maintain and widen the spotlight on DEI topics. Pooling together the efforts of many nucleating agents, we will reach a critical mass to bring about change from the inside our own communities.

In our experience, most plant biologists support the goal of a more inclusive discipline, but many find it challenging to participate in what can be difficult conversations around race, ethnicity, gender identity, sexuality, and ability. In difficult dialogues, it is often helpful to assume good intentions. Instead of publicly accusing individuals of deliberately causing harm, one might first privately and gently highlight how their words or actions could be perceived. Survival bias and reluctance to change can be significant obstacles, so an approach focused on ‘calling in’ rather than ‘calling out’ offenders (Herbison and Podosky, 2024) can reduce defensiveness.

A significant concern raised during the Q&A session at the end of the workshop was whether holding such a workshop is merely preaching to the converted. Concurrent and optional sessions tend to self-select for interested individuals, and those that might benefit the most from discussions about DEI might not be in those rooms. Highlighting research culture topics during conference plenaries or in regular internal institute seminars is one solution. Other dissemination routes should also be pursued; with this article—for example—we hope to reach a wider audience than that physically present in our original workshop.

The culture of science has been studied for decades by social scientists, who have published articles and books documenting and charting a path forward to greater inclusivity (some of which have been referenced in this piece). We encourage institutions and organizations to leverage their power to expose biological scientists to these important works, help to scaffold discussions, and highlight the value of inclusive practices. Responding to claims of science ‘objectivity’ with data-driven and factual evidence of the opposite is powerful. The support of social practitioners embedded in these topics can also reduce feelings of imposter syndrome by biologists actively trying to promote positive DEI practices.
